# Editorial: Clinical trials in drug metabolism and transport: 2022

**DOI:** 10.3389/fphar.2023.1223428

**Published:** 2023-06-13

**Authors:** Yurong Lai, Stanislav Yanev, Zhihao Liu

**Affiliations:** ^1^ Gilead Sciences Inc., Foster City, CA, United States; ^2^ Institute of Neurobiology, Bulgarian Academy of Sciences (BAS), Sofia, Bulgaria; ^3^ Department of Clinical Pharmacology, College of Pharmacy, Dalian Medical University, Dalian, China

**Keywords:** clinical trials, pharmacoknietics, metabolism, transport, physiologically based pharmacokinetic (PBPK)

Investigational drugs that have been demonstrated reasonably safe and efficacious for disease therapy can be tested in humans to elucidate the pharmacokinetic (PK) behavior, pharmacodynamics (PD), safety, tolerance, and efficacy in healthy volunteers and subjects suffering from diseases (Mauri et al., 2018). Human clinical trials are an essential component of the drug development process, as they provide critical information about a new drug’s safety, efficacy, and mechanism of action. The practices are irreplaceable for obtaining valuable insights into specific questions. They serve as an essential educational resource for further studies and comparisons. The processes must be included, as the PK data are necessary for determining appropriate dosing regimens, informing the drug interactions, and establishing the relationship between PK and efficacy and safety.

High-quality data obtained in clinical trials can be used in understanding how the body processes a drug, including how it is absorbed, distributed, metabolized, and excreted (ADME). Publishing these high-quality original data can significantly advance the field and provide a basis for developing new drugs or improving existing ones (Li et al., 2019). In addition, it is known that metabolism and transport are two crucial components of PK. As expected, clinical trials in PK are critical in advancing our understanding of how drugs are metabolized and transported in the body and developing safe and effective patient medications.

Conventional scientific journals typically prioritize the publication of research demonstrating significant positive outcomes or insights into the mechanisms underlying a drug’s effects. However, clinical trial data sometimes need more positive results and mechanism insights, making publishing the findings in a scientific journal challenging. When clinical trial data fails to meet these criteria, it may be rejected for publication or remain unpublished, leaving the results inaccessible to the scientific community and the public. In some cases, these negative or inconclusive clinical trial results may become available to the public through other means, such as public registries or reports fields with regulatory agencies. However, with the context and analysis provided by peer-reviewed scientific publications, it can be easier for individuals outside the scientific community to fully interpret and understand these results.

In recent years, there has also been a growing push for greater transparency and access to clinical trial data, regardless of the outcomes. Many researchers and organizations advocate for the publication of all clinical trial results, regardless of whether they demonstrate positive outcomes or insights, to ensure that the scientific community and the public have access to the full range of information about a drug’s effects and potential risks.

The current Research Topic accepts traditional aspects of drug-metabolizing enzymes, drug transporters, and complementary facets critical for a clear understanding of the current field and upcoming challenges. Finally, a total of 12 original articles were published on this Research Topic, covering several aspects related to this field, including the bioequivalence trials, phase I clinical trials in healthy subjects, population PK analysis in patients, physiologically based pharmacokinetic (PBPK) modeling and simulation study, and drug-drug/food interaction evaluation.

Understanding the PK and tolerability of drugs in healthy volunteers is a critical step in drug development, which can provide valuable information on drug pharmacokinetics and ADME behaviors. After confirming that a new investigational drug is reasonably safe to dose in humans, Phase I clinical trials can be followed for the human safety, tolerability, PK and PD of a new investigational drug in a small number of healthy volunteers or patients with the disease of interest. The maximum tolerated dose MTD or efficacious phase II exposure of the investigational drugs can be determined in subsequent efficacy and safety studies. Phase I trials may also provide preliminary data on the drug’s efficacy, optimal dosing regimen, and potential biomarkers for monitoring the drug’s activity. On the other hand, human bioequivalence studies administer two drug products to healthy volunteers or patients in a randomized crossover design to determine whether the test product meets the regulatory criteria for bioequivalence. The data usually expressed in terms of confidence intervals for vital pharmacokinetic parameters such as the area under the curve (AUC) and maximum concentration (Cmax) are critical for regulatory authorities in approving and regulating generic drugs. Qu et al. and others conducted a phase I bioequivalence trials to compare cefaclor granule and cefaclor suspension were bioequivalent in healthy Chinese subjects in fasting and postprandial states. The authors found that both Cefaclor granule and Cefaclor suspension are bioequivalent with similar PK profiles, and food effects are observed for both formulations.

To assess the PK, safety, and tolerability profiles of a potential selective JAK1 inhibitor, WXFL10203614, Huang et al. and others conducted a randomized, open-label, crossover study in 14 healthy subjects receiving single and multiple oral doses of WXFL10203614. The authors found that WXFL10203614 showed good tolerability and favorable PK and safety profiles in healthy Chinese subjects, which supports further clinical development in patients with rheumatoid arthritis. A follow-up trial conducted by Huang et al. investigated the food effects on the PK of the drug candidate WXFL10203614. The authors found that WXFL10203614 is rapidly absorbed in fasted conditions. The high-fat and high-calorie diet intake can lower its absorption rate; however, the PK changes are not clinically relevant.

101BHG-D01 is a selective muscarinic receptor antagonist with stable physical-chemical properties and is developing for treating chronic obstructive pulmonary disease. Gao et al. conducted a randomized, double-blind, placebo-controlled dose-ranging finding study of 101BHG-D01 inhalation aerosol to evaluate its pharmacokinetics, metabolite profiling, safety, and tolerability in healthy Chinese subjects. The authors observed no circulating metabolites at greater than 10% of total drug-related exposure. The results indicate that 101BHG-D01 can be a suitable candidate for further clinical development in subsequent studies in COPD patients.

SHR2285 is a selective human coagulation factor XIa inhibitor and is a potential anticoagulant. After completing three clinical phase I trials in healthy subjects, Ma et al. conducted a single-center, randomized, double-blind, placebo-controlled studies to assess its safety, tolerability, PK, and PD in combination with aspirin, clopidogrel or ticagrelor. The authors observed the dose-dependent inhibition of FXIa activity with SHR2285. In healthy subjects, co-administration of aspirin, clopidogrel, or ticagrelor with SHR2285 was well-tolerated, and no safety or tolerance concerns were identified. Meanwhile, these authors also published a randomized, double-blind, single-dose escalation, placebo-controlled phase I trials to evaluate the safety, tolerability, PK, and immunogenicity of an anti-nerve growth factor monoclonal antibody candidate for treating pain, DS002 injection, in healthy Chinese subjects. Based on the results, the authors concluded that DS002 demonstrated good safety profiles within the tested dose ranges and, through blocking nerve growth factor, it is expected to be a novel, safe and non-addictive treatment for pain.

Besides single-dose administration, clinical trial studies in PK may involve the administration of multiple doses of a drug to evaluate its behavior (Kasichayanula et al., 2018). In a 7-days repeating dosing study, Khan et al. investigated the comparative tolerability and metabolism of primaquine, an 8-aminoquinoline antimalarial, in normal glucose-6-phosphate dehydrogenase (G6PDn) and G6PD deficient subjects (G6PDd). Primaquine is a racemic drug, and the enantiomers have widely divergent metabolites and exposure to the parent drug. Both enantiomers and racemates consistently showed gradual increases in methemoglobin and bilirubin. However, in a single hemizygous G6PDd male, the bilirubin response was much more pronounced and required discontinuation of both enantiomers.

Population PK models are a branch of PK that aims to describe and quantify the variability in drug PK among individuals within a population (Klünder et al., 2018). It uses mathematical models to analyze data from large patient groups to understand better how drug concentrations in the body change over time. Li et al. and others built a nonlinear mixed-effect population PK model to analyze PK variables in patients with solid tumors of TQ-B3203, a novel topoisomerase I inhibitor currently developing for the treatment of advanced solid tumors. The authors found that direct bilirubin and body mass index (BMI) were the two most influential factors in clearance. The model can aid the design of future clinical trials by optimizing the dose regimen for TQ-B3203. Similarly, Luo et al. developed a non-linear mixed-effect population PK model for tigecycline, a new type of antibiotic, in critically ill patients. The model suggested that APACHEII score and age are two variables to impact the clearance and volume distribution of tigecycline, respectively. The standard dosing schedule of tigecycline may not be optimal for sound therapeutic effects for critically ill patients.

Physiologically based pharmacokinetic (PBPK) modeling is a mathematical modeling technique used to predict the pharmacokinetic behavior of drugs in the human body based on physiological and anatomical parameters. The model divides the human body into compartments representing different tissues or organs and then uses mathematical equations to describe how the drug moves between these compartments. PBPK modeling has increased in recent years to evaluate the impact of factors such as age, sex, disease state, and drug-drug interactions on drug exposure. It can aid in predicting the optimal dosing regimen in various populations (Smits et al., 2019). In this Research Topic, Li et al. developed a PBPK model to predict the plasma concentration profiles of schaftoside, a significant ingredient of traditional Chinese medicine for treating urolithiasis. The model was first built using *in vivo* rat data following iv and oral administration of the total flavonoids and extrapolated to humans through incorporating *in vitro* human data. The PBPK model helps determine appropriate dosages of the total flavonoids of Desmodium styracifolium in various populations, representing a practical approach for evaluating the effectiveness and safety of herbal remedies.

A radioactive mass balance study is a type of clinical trial that involves administering a small amount of radiolabeled drug to humans and measuring the amount of radioactivity in various biological excreta over some time, to determine the fate of the drug in the body, including its ADME pathways. This type of study is beneficial for evaluating the ADME properties of new drugs or for comparing the ADME properties of different formulations of the same drug. Zhang et al. investigated the PK, biotransformation pathway, and mass balance of [14C]SHR6390, a selective and effective cyclin-dependent kinase 4/6 inhibitor, in humans. The results showed that 94.63% of the dose is recovered in the urine and feces after a single oral administration of [14C] SHR6390 and fecal elimination is a major route. Also included in this Research Topic, Guo et al. investigated the PK behavior in healthy beagles receiving single and multiple administrations of glutamine tablets developed for potential veterinary use to treat animal gastrointestinal diseases. The study characterized the ADME properties of glutamine tablets in dogs and discovered the PK parameters and dose linearity. The results provided a theoretical guide for pet clinical practice and formulation development.

Overall, the current research focuses on showcasing top-tier clinical trials published in Drug Metabolism and Transport, encompassing traditional aspects of drug-metabolizing enzymes and drug transporters and critical complementary facets contributing to a comprehensive understanding of the current landscape and future challenges. It provides a valuable platform for publishing high-quality clinical trials and promoting discoveries in the field. Drug hunters can use the data published to develop safer and more effective patient medications by advancing our understanding of these critical areas.
